# The Right Ventricle in Cardiac Critical Care: Pathophysiology, Evaluation and Management

**DOI:** 10.3390/medicina62061070

**Published:** 2026-06-01

**Authors:** Aristi Boulmpou, Ioannis Alevroudis, Efstratios Karagiannidis, Sophia-Anastasia Mouratoglou, Athina Nasoufidou, Nikolaos Fragakis, Christodoulos Papadopoulos, Vassilios Vassilikos

**Affiliations:** 1 Third Department of Cardiology, Ippokratio General Hospital, Aristotle University of Thessaloniki, 546 42 Thessaloniki, Greece; 2 Department of Emergency Medicine, AHEPA University Hospital, 546 36 Thessaloniki, Greece; 3 Second Department of Cardiology, Ippokratio General Hospital, Aristotle University of Thessaloniki, 546 42 Thessaloniki, Greece

**Keywords:** right ventricle, cardiogenic shock, cardiac care unit, cardiovascular imaging, mechanical circulatory support

## Abstract

The right ventricle (RV) is a primary determinant of outcomes in cardiac critical care. RV dysfunction independently predicts morbidity and mortality in conditions such as acute coronary syndromes, pulmonary embolism, and cardiogenic shock. This review synthesizes RV evaluation and management by integrating physiologic principles with bedside diagnostic and therapeutic strategies. The RV is exceptionally sensitive to acute afterload increases due to its adaptation to low-pressure pulmonary circulation. Evaluation utilizes a multimodal approach combining echocardiography, invasive hemodynamics, and specifically the pulmonary artery pulsatility index and central venous pressure/pulmonary capillary wedge pressure (CVP/PCWP) ratio and biomarkers. Management focuses on three pillars: individualized preload optimization, afterload reduction via selective pulmonary vasodilators, and contractility augmentation with inotropes. For refractory cases, mechanical circulatory support options like Impella RP, ProtekDuo, and VA-ECMO provide critical bridges to recovery or transplantation.

## 1. Introduction

The right ventricle (RV), once considered the “forgotten chamber,” has increasingly emerged as a central determinant of outcomes in cardiac critical care [1]. Historically, clinical and research attention has focused predominantly on the left ventricle (LV), with the RV long regarded as a secondary chamber in cardiac performance [2]. However, accumulating evidence over recent decades has highlighted that RV dysfunction independently predicts morbidity and mortality across a wide spectrum of cardiovascular and critical care conditions, from acute coronary syndromes (ACS) and pulmonary embolism (PE) to cardiogenic shock (CS) and advanced heart failure (HF) [3,4].

Renewed attention to the RV reflects a growing appreciation of its unique physiology and its major influence on hemodynamic stability in critical illness [5]. Contemporary cardiac care units (CCUs) are increasingly managing older, more complex patients, often with multiple comorbidities and a need for advanced hemodynamic support. Advances in echocardiography and invasive monitoring now allow more precise assessment of RV structure and performance, while emerging imaging modalities and biomarkers further expand diagnostic capabilities [6,7]. Yet, despite these developments, the integration of RV evaluation into routine decision-making remains inconsistent.

This review distinguishes itself from existing literature by providing a contemporary synthesis that bridges the gap between traditional physiologic principles and the rapidly evolving technological landscape of the modern CCU. While previous reviews often treat imaging and hemodynamics in isolation, this article integrates advanced phenotyping with the routine application of the pulmonary artery pulsatility index (PAPi) and the ratio of central venous pressure to pulmonary capillary wedge pressure (CVP/PCWP) to guide the timing of intervention. By doing so, it offers a specific framework for the “early-exit” strategy, supporting the prompt initiation of temporary mechanical circulatory support (MCS) to prevent multi-organ failure rather than relying on prolonged pharmacological treatments. Furthermore, the discussion of emerging tools like artificial intelligence and refined risk scores is intended to offer a forward-looking perspective on real-time bedside decision support in an increasingly complex patient population.

## 2. Pathophysiology of RV Dysfunction in Cardiovascular Disease

**Figure 1:**
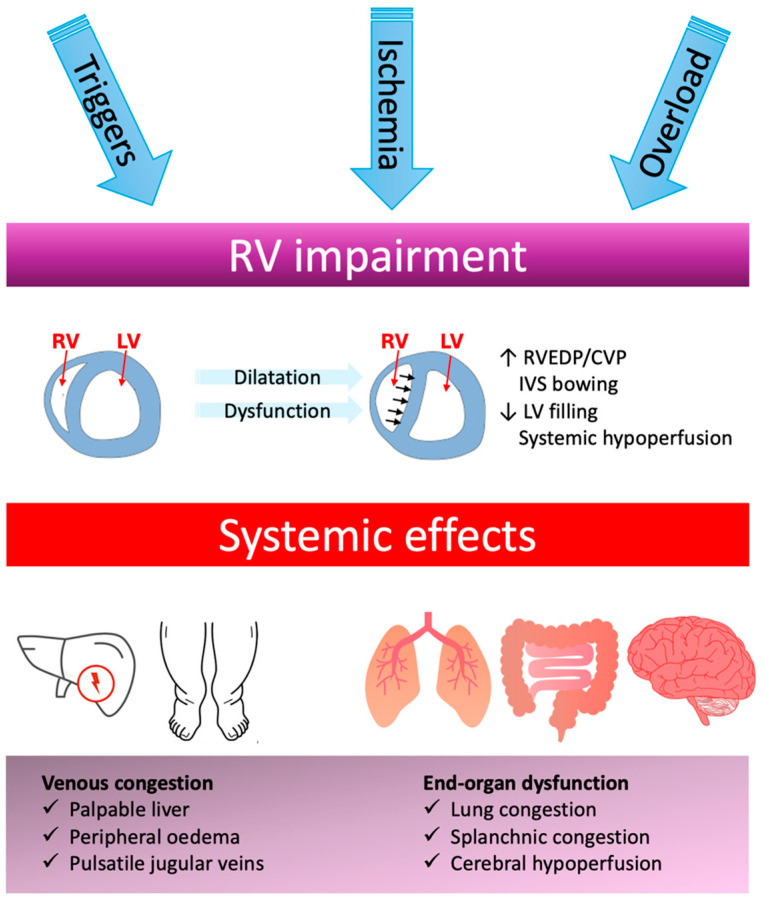
Pathophysiology of right ventricular dysfunction in cardiac critical care. Schematic overview linking mechanisms of right ventricular (RV) dysfunction to hemodynamic consequences and bedside management strategies. Acute RV injury arises predominantly from ischemia, pressure overload, or volume overload, often in combination. These mechanisms lead to RV dilation, reduced contractility, interventricular septal shift, and impaired RV–pulmonary artery coupling, resulting in decreased left ventricular preload, systemic hypotension, and venous congestion with end-organ dysfunction. Abbreviations: RV, right ventricular; LV, left ventricular; RVEDP, right ventricular end-diastolic pressure; CVP, central venous pressure.

The RV is anatomically and functionally distinct from the LV, featuring a thin free wall and crescentic geometry adapted for the high-compliance pulmonary circulation [8,9]. While preload-dependent, the RV has limited reserve; excessive volume leads to dilation, increased wall stress, and reduced contractile efficiency [10]. The RV is particularly sensitive to afterload, where even small increases in pulmonary vascular resistance (PVR) can disproportionately reduce stroke volume [11,12]. Ventricular interdependence further influences performance; as the RV dilates, the interventricular septum shifts to the left, impairing LV diastolic filling and decreasing systemic cardiac output (Figure 1) [11,13].

Beyond these individual mechanisms, modern RV physiology unifies ischemia, pressure, and volume overload under a systems-level model of RV–pulmonary artery (PA) coupling. The gold standard for this relationship is the coupling ratio of RV end-systolic elastance (E_es_, contractility) to pulmonary arterial elastance (E_a_, afterload), where an acute ratio < 1.0 signifies mechanical uncoupling. This uncoupling drives a dual-phenotype cascade: a forward failure phenotype resulting from severe oxygen supply-demand mismatch and energetic failure and a backward failure phenotype characterized by systemic venous congestion that causes renal and hepatic injury [14].

This energetic failure is further driven by a distinct metabolic phenotype that casts the RV uniquely vulnerable compared to the LV. At baseline, the RV myocardium possesses a lower mitochondrial density and a reduced capillary-to-myocyte ratio than the LV [15]. Consequently, when subjected to acute pressure overload, the RV is highly susceptible to profound oxidative stress, impaired fatty acid oxidation, and an abrupt glycolytic shift. These intrinsic metabolic constraints accelerate the progression of RV failure under stress, long before similar loading conditions would compromise LV function.

### 2.1. Ischemic Injury

RV ischemia represents an important but often underrecognized mechanism of dysfunction in critical care. Although the RV has lower oxygen demand than the LV and receives coronary perfusion during both systole and diastole, it is highly dependent on adequate coronary perfusion pressure [9,16]. Systemic hypotension, elevated RV end-diastolic pressure, and tachycardia can all compromise RV myocardial blood flow.

Ischemia may result from acute right coronary artery occlusion, global hypoperfusion in shock states, or severe hypoxemia. Importantly, RV ischemia may also occur due to pressure or volume overload, in which increased wall tension raises myocardial oxygen demand while simultaneously reducing subendocardial perfusion [12,17]. This mismatch establishes a self-perpetuating cycle of worsening contractile dysfunction and hemodynamic collapse [3,18].

At the cellular level, acute decompensation triggers a profound disruption in RV energetics and metabolism. Under sustained pressure or ischemic stress, the RV myocardium undergoes a metabolic shift, transitioning from its highly efficient baseline fatty acid oxidation to a state of accelerated glycolysis. This metabolic reprogramming is accompanied by progressive mitochondrial dysfunction and the rapid accumulation of reactive oxygen species, which induces oxidative stress and impairs cellular viability [19]. Ultimately, this energetic crisis leads to calcium handling dysregulation within the cardiomyocytes, disrupting excitation–contraction coupling and accelerating the transition from adaptive hypertrophy to overt contractile failure [20].

### 2.2. Pressure Overload

Acute RV pressure overload is a dominant cause of RV failure in the CCU and exemplifies the RV’s vulnerability to abrupt increases in afterload [18]. Acute pulmonary embolism is the paradigmatic condition, where sudden obstruction of the pulmonary vasculature leads to a rapid rise in PVR. The RV responds with dilation, reduced systolic shortening, and increased wall stress, often accompanied by interventricular septal shift and impaired LV filling [21].

Other critical care scenarios associated with RV pressure overload include acute exacerbations of pulmonary hypertension, hypoxic pulmonary vasoconstriction in acute respiratory distress syndrome, and the hemodynamic effects of positive-pressure ventilation. Unlike chronic pressure overload, which may allow time for adaptive hypertrophy, acute increases in afterload overwhelm the RV’s limited contractile reserve, precipitating rapid decompensation [3,18,22].

This elevation in afterload extends beyond a simple mechanical increase in pulmonary PVR. At the cellular level, it is driven by acute disruptions in pulmonary vascular biology, including endothelial dysfunction characterized by an imbalance between vasodilators and vasoconstrictors and, specifically, a reduction in nitric oxide bioavailability and an upregulation of endothelin-1 [23]. Furthermore, this process is exacerbated by hypoxic pulmonary vasoconstriction pathways that acutely increase smooth muscle tone, which, in chronic states, transitions into structural vascular remodeling of the pulmonary arterioles [24].

### 2.3. Volume Overload

RV volume overload arises from conditions that increase RV preload or result in regurgitant flow, most commonly severe tricuspid regurgitation. Acute volume loading leads to RV dilation, elevated wall stress, and reduced mechanical efficiency. As the RV enlarges, pericardial constraint and septal displacement further impair LV diastolic filling, compounding systemic hypotension [11].

In the critical care setting, RV volume overload may also result iatrogenically, related to aggressive fluid resuscitation in shock. While chronic volume overload may be initially tolerated through chamber dilation and remodeling, acute illness often unmasks limited RV reserve, leading to overt failure.

Regardless of the initiating mechanism, RV dysfunction produces a characteristic cascade of hemodynamic and clinical consequences. Reduced RV stroke volume limits pulmonary blood flow and LV preload, resulting in decreased systemic cardiac output and hypotension. Simultaneously, elevated right-sided filling pressures cause systemic venous congestion [18,25].

Venous congestion has emerged as a central contributor to end-organ dysfunction in critical illness. Increased renal venous pressure impairs glomerular filtration and promotes acute kidney injury, while hepatic congestion leads to hepatocellular dysfunction and cholestasis. Gastrointestinal edema, impaired drug absorption, and ascites further complicate management. In advanced stages, RV failure culminates in cardiogenic shock with lactic acidosis and multiorgan failure [18,25].

There is evidence demonstrating that RV dysfunction has major prognostic significance across different critical care populations. In acute pulmonary embolism, echocardiographic or biomarker evidence of RV strain identifies patients at increased risk of hemodynamic collapse and mortality, even in the absence of systemic hypotension [21]. In septic shock, RV dysfunction is common and independently associated with worse outcomes, reflecting the combined effects of myocardial depression, pulmonary vascular abnormalities, and mechanical ventilation [26].

In acute and chronic HF, RV function is a key determinant of symptoms, response to therapy, and survival. The presence of RV dysfunction identifies a high-risk phenotype characterized by venous congestion, limited cardiac reserve, and poor tolerance of standard therapies [13,18]. Similarly, in cardiogenic shock, impaired RV performance predicts failure of pharmacologic support and adverse outcomes with MCS [27].

While ischemia, pressure overload, and volume overload can initiate RV injury independently, they rarely exist in isolation during critical illness. Instead, they merge into a single hemodynamic progression model. An acute increase in pressure or volume overload immediately elevates RV wall stress, which raises myocardial oxygen demand. This increased demand occurs simultaneously with a drop in right coronary artery perfusion pressure caused by systemic hypotension, precipitating secondary ischemic injury. This ischemia further impairs contractility, worsening chamber dilation and septal shift, which ultimately accelerates forward and backward pump failure.

## 3. Evaluation of the Right Ventricle in Critically Ill Patients

The assessment of RV function in the critical care setting represents a major diagnostic challenge due to the chamber’s unique anatomy and physiology. The RV is characterized by a complex, crescent-shaped geometry that wraps around the LV, making it difficult to model with simple geometric assumptions [6]. Furthermore, the performance of the RV is highly load-dependent; contractility can be easily impaired or exaggerated by significant fluctuations in preload and afterload, which are far more common among critically ill patients [28]. Consequently, a single parameter for the evaluation of RV systolic and diastolic performance is rarely sufficient. A robust evaluation includes a multimodal approach that integrates bedside imaging with hemodynamic data to accurately phenotype the patient’s condition. 

### 3.1. Echocardiography

Transthoracic echocardiography (TTE) remains the cornerstone of RV assessment in the acute setting due to its portability, safety, and reproducibility [29]. In daily clinical practice, the initial assessment relies on conventional indices of longitudinal function, such as tricuspid annular plane systolic excursion (TAPSE), with a value of <17 mm indicating dysfunction, and the systolic excursion velocity of the tricuspid annulus (s’), where a velocity < 9.5 cm/s suggests impaired performance [30]. These parameters are widely validated and easy to obtain, making them ideal for identifying gross dysfunction. However, they are limited by angle dependency and the fact that they primarily measure the displacement of the basal segment, potentially overestimating global function in patients with regional wall motion abnormalities [31]. RV fractional area change (FAC) provides a more comprehensive estimate of RV systolic function by incorporating radial contraction, but its accuracy depends highly on endocardial border definition, which is often suboptimal in mechanically ventilated patients [32].

When standard echocardiographic windows are inadequate, a rather frequent scenario among CCU patients due to mechanical ventilation, surgical dressings, or body shape, transesophageal echocardiography (TEE) becomes an essential “rescue” modality. TEE provides superior visualization of the right heart structures that are often obscured on TTE, particularly the right atrial appendage, the superior vena cava, and the RV outflow tract (RVOT) [33]. TEE is particularly useful for excluding intracardiac thrombi in pulmonary embolism or for guiding cannula positioning in patients requiring MCS [34]. However, TEE is invasive and requires specific operator expertise, reserving it for cases when TTE is non-diagnostic or when precise procedural guidance is required.

To improve diagnostic sensitivity, contemporary practice integrates advanced echocardiographic techniques that overcome the geometric limitations of 2D assessments. RV longitudinal strain via speckle tracking has emerged as a superior metric, offering higher sensitivity for detecting early, subclinical dysfunction and providing independent prognostic value in heart failure and pulmonary hypertension [35,36]. In the same line, three-dimensional (3D) echocardiography allows for the precise volumetric assessment of the RV and the calculation of the RV ejection fraction (RVEF) without the need for geometric assumptions, which is a significant advancement over 2D area-based estimates. While these advanced modalities correlate strongly with reference standards, their application in the acute setting is often restricted by the requirement for stable heart rates and high-quality images, which are frequently unavailable in hemodynamically unstable patients [37].

### 3.2. Invasive Hemodynamics

When non-invasive imaging is inconclusive, pulmonary artery catheterization is essential to characterize RV–pulmonary coupling [38]. CVP alone is considered an unreliable indicator of RV function [39]. Clinicians should instead prioritize the PAPi, where low values are strongly associated with severe RV dysfunction and help guide the timing of MCS; a value < 1.0 in the setting of acute myocardial infarction or <1.85 post-LV assist device (LVAD) implantation is highly suggestive of RV failure. Furthermore, the CVP/PCWP ratio helps differentiate between biventricular failure and predominant right heart failure; a ratio exceeding 0.8 typically identifies primary RV dysfunction, allowing for tailored RV offloading rather than generic HF management [40].

### 3.3. Complementary Modalities: Imaging and Biomarkers

Beyond echocardiography and hemodynamics, cardiac magnetic resonance (CMR) is established as the reference standard for the precise quantification of RV volumes and mass due to its superior spatial resolution and comprehensive anatomical coverage [41]. Its ability to provide accurate data without relying on geometric assumptions makes it the definitive tool for assessing RV performance. However, CMR is often logistically unfeasible in the acute phase for hemodynamically unstable patients due to the requirements of the examination environment and long acquisition times. Consequently, its primary role remains tissue characterization and gold-standard quantification in stable patients to aid in the diagnosis of specific etiologies such as myocarditis or arrhythmogenic cardiomyopathy [42].

In the acute setting, computed tomography (CT) is frequently utilized, primarily to exclude pulmonary embolism. However, modern gated cardiac CT protocols offer the added benefit of assessing RV geometry and size in patients with poor echocardiographic windows, providing a crucial alternative for anatomical assessment [43]. Finally, cardiac biomarkers such as B-type natriuretic peptides and high-sensitivity cardiac troponins serve as sensitive indicators of myocardial wall stress and injury. While non-specific, a disproportionate elevation of these biomarkers in the setting of acute RV strain, such as in pulmonary embolism, carries significant prognostic weight and should prompt immediate escalation of diagnostic and therapeutic efforts [44]. Ultimately, these biomarkers should not be viewed as standalone findings, but rather integrated directly into clinical risk-stratification models alongside imaging data to identify high-risk phenotypes prone to rapid hemodynamic collapse.

## 4. Most Common Clinical Scenarios Where RV Evaluation Is Crucial

### 4.1. Acute Coronary Syndromes with Possible RV Involvement

RV involvement occurs in approximately 50% of inferior ST-elevation myocardial infarctions (STEMIs) and is associated with increased morbidity, mortality, and late adverse major adverse cardiovascular events (MACEs) [45]. Despite its clinical impact, RV infarction is frequently underdiagnosed because right precordial leads are not systematically recorded in standard practice. To ensure timely detection, it is recommended to routinely obtain right-sided ECG leads, specifically V3R and V4R, in all cases of inferior STEMI. The classic clinical presentation of hypotension and elevated jugular venous pressure in the presence of clear lungs should immediately prompt this diagnostic step.

The RV is less susceptible to ischemia compared to the LV due to lower oxygen requirements and the ability to be reperfused during both systole and diastole. RV dysfunction leads to reduced LV preload and systemic hypotension, typically without pulmonary congestion, creating a challenging clinical scenario for the treating physician [4]. RV systolic performance relies greatly on the interventricular septal motion and on the right atrial (RA) contraction. Proximal occlusion of the right coronary artery may compromise right atrial perfusion, thereby eradicating an important aspect of the RV systolic function [46]. RA ischemia may further cause rate and rhythm disturbances like high-degree atrioventricular (AV) block secondary to AV nodal ischemia and atrial fibrillation (AF) due to increased RA wall stress and pressures [47].

In addition, RV function is closely associated with vagally mediated bradyarrhythmias, which may occur both during the acute ischemic phase and following reperfusion. The autonomic system instability is largely affected by pain and hypotension. Conduction delays change the electrical homeostasis, facilitating ventricular tachycardia (VT) [48]. RV ischemia and infarction can also lead to acute RV dilatation. Stretching of the myocardial fibers alters ion channel behavior, resulting in enhanced triggered activity and re-entry, predisposing patients to life-threatening VT [49].

### 4.2. Pulmonary Embolism

The RV is the major cardiac chamber affected in PE. PE leads to an acute increase in pulmonary arterial pressure and RV afterload due to mechanical obstruction by emboli and the release of constrictive mediators [50]. Due to its thin wall, the RV is poorly adapted to sudden pressure overload, resulting in acute RV dilatation and a subsequent ventricular septal shift, as described in the general pathophysiology section [51]. This process can lead to a life-threatening vicious cycle of progressive RV dilatation and worsening ischemia.

The presence and severity of RV dysfunction are key determinants of risk stratification. The pulmonary embolism severity index (PESI), which predicts 30-day mortality after acute PE, incorporates variables such as high heart rate, hypotension, and oxygen saturation, all of which are clinical manifestations that largely reflect underlying RV dysfunction and contribute to increased mortality [52]. Moreover, the presence of hypotension or shock alone defines PE as high-risk for early mortality, while RV dysfunction and PESI are two major contributors to adverse outcomes [21]. However, caution is mandatory even for intermediate-risk patients, as one-third of them present with normotensive shock [53].

RV impairment can also cause long-term consequences. Post-pulmonary embolism syndrome is defined as new or progressive shortness of breath, exercise intolerance, and/or impaired functional or mental status, with increased prevalence observed among PE patients [54].

### 4.3. Cardiogenic Shock

Even though cardiogenic shock due to RV failure is less recognized in the literature, the most common causes are PE, inferior myocardial infarction, and RV infarction. Patients are usually of a younger age, with a higher mortality risk compared to cardiogenic shock due to LV failure. The basic principles remain a systolic blood pressure of 90 mmHg and clinical and laboratory evidence of end-organ damage (i.e., urine output < 0.5 mL/kg or serum lactate > 2 mmol/L) [54]. RV shock typically presents with hypotension, increased jugular venous pressure, and clear lungs, reflecting systemic venous congestion rather than pulmonary edema [55]. In contrast, LV shock presents with pulmonary congestion and low cardiac output, with lower right-sided filling pressures [56]. This diversity helps the clinician differentiate the underlying cause of shock and change the therapeutic management accordingly.

Echocardiography and invasive monitoring can aid in this challenging diagnosis. RV shock requires volume optimization, inotropes, and peripheral vascular resistance decline, while LV shock requires aggressive diuresis, vasodilators, and systemic vascular resistance reduction. RV dysfunction is often associated with poor outcomes and has been used as a prediction tool for mortality [57]. When pharmaceutical options fail, bailout strategies with assistive devices can be utilized [58].

### 4.4. Pulmonary Arterial Hypertension

RV function is the major determinant of symptoms, functional status, and survival in patients with pulmonary arterial hypertension (PAH). The ability of the RV to adapt to changes in the pulmonary vasculature, the gradual increase in PVR, and the resulting chronic pressure overload is a key predictor of mortality [59]. RV dilatation, systolic dysfunction, increased atrial pressures, and reduced cardiac output often precede right HF, which is the leading cause of mortality in patients with PAH. Moreover, hemodynamic parameters like mean pulmonary artery pressure (mPAP), RA pressure, and cardiac output are key predictors of prognosis, all related to the RV [60,61]. Non-invasive imaging, including echocardiography and CMR, but also right heart catheterization, is essential for the assessment of RV function and structure [60]. Therapeutic management in PAH is focused not only on decreasing pulmonary vascular resistance (PVR) but also on preserving RV function, highlighting the importance of the RV in this clinical setting [61].

### 4.5. Advanced Heart Failure

RV function is a major component of the overall cardiac performance in advanced heart failure (AHF). In this setting, the RV is enlarged, with reduced systolic function related to myocardial fibrosis [62]. Isolated RV failure, impaired exercise capacity indicated by a declined peak oxygen uptake (peak VO2), and liver dysfunction as evidence of end-organ damage are elements of AHF that require referral to an expert center for further management, which includes heart transplant or an LVAD implantation [63]. In studies of patients with AHF, RV dysfunction has been consistently correlated with poor outcomes and reduced survival. RV indices, like the RV myocardial performance index or RV free wall strain, have been utilized for prognostic information and risk stratification [64,65]. Moreover, improvement of RV mechanics after intensification of medical therapy has been associated with lower long-term adverse events in patients with acute decompensation [66]. Arrhythmic aspects of AHF are often underestimated; however, RV function is highly dependent on a regular cardiac rhythm and should be carefully considered in both assessment and management [67,68].

**Figure 2:**
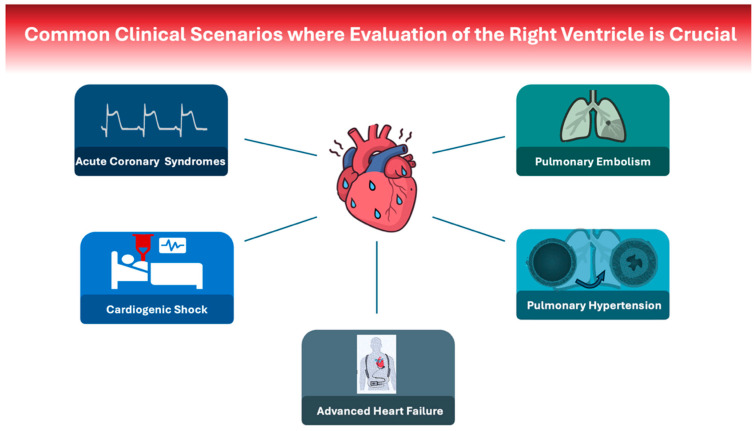
Common clinical scenarios where evaluation of the right ventricle is crucial.

Figure 2 summarizes the most common clinical scenarios where the evaluation of the RV is of paramount importance.

## 5. Management Principles

### 5.1. Hemodynamic Optimization

Acute RV failure management in the CCU represents a significant clinical challenge requiring a deep understanding of RV physiology and its unique response to hemodynamic changes. Management is directed at three fundamental goals: RV preload optimization, RV afterload reduction, and augmenting contractility. Unlike the LV, the thin-walled, compliant RV is exceptionally sensitive to acute elevations in PVR. The pathophysiology of decompensation involves a vicious cycle where afterload-induced mechanical uncoupling elevates wall tension and oxygen demand while decreasing coronary perfusion pressure, rapidly predisposing the RV to ischemia [69].

The presented management framework emphasizes a shift away from traditional, aggressive volume-loading strategies in favor of dynamic parameters and the prevention of congestive end-organ injury. This approach integrates a targeted view of RV-PA coupling with the use of selective pulmonary vasodilators and specific MCS platforms, such as the Impella RP and ProtekDuo, to navigate the complex cycle of RV failure.

### 5.2. Preload Optimization and Fluid Management

A radical change has occurred regarding fluid management, moving away from aggressive volume loading. While the failing RV requires adequate preload to maintain cardiac output according to the Frank–Starling relationship, excessive volume can be severely damaging. In acute RV failure, most patients are not preload-dependent; instead, function is exacerbated by volume overload. Increased preload further distends the RV, worsens tricuspid regurgitation, and shifts the septum leftward, ultimately impairing LV filling and systemic output [69].

Early clinical assessment of volume status is paramount. As CVP has significant limitations as a predictor of fluid responsiveness, dynamic parameters, including stroke volume variation (SVV), pulse pressure variation (PPV), and passive leg raising, provide more reliable evaluations. Echocardiographic evaluation of RV size, septal motion, and inferior vena cava (IVC) dynamics offers real-time guidance. When hypovolemia is suggested, a cautious fluid challenge of 250–500 mL can be considered. However, in patients with venous congestion, aggressive diuresis with loop diuretics is the priority to restore the perfusion pressure gradient.

### 5.3. Afterload Reduction Strategies

Reducing RV afterload is a cornerstone of management, particularly in acute cor pulmonale, where elevated PVR is the primary driver [70]. Oxygen acts as a potent pulmonary vasodilator, and aggressive therapy can reduce PVR by up to 25% [71]. Correction of acidemia and avoidance of hypercapnia are equally essential, as both augment hypoxic pulmonary vasoconstriction.

Mechanical ventilation profoundly affects RV function; while positive pressure reduces RV preload, high airway pressures increase PVR by compressing alveolar vessels. “RV-protective ventilation” emphasizes plateau pressure limitation to mitigate these effects [72].

### 5.4. Pharmacologic Considerations

Systemic hypotension demands prompt treatment to maintain coronary perfusion pressure to the RV free wall. The goal is to maintain systemic arterial pressure higher than pulmonary arterial pressure.

• Vasopressors: Norepinephrine is the preferred first-line agent. Through alpha-1 receptor stimulation, it provides potent systemic vasoconstriction, while its modest beta-1 effect restores RV-PA coupling better than pure inotropy [73]. Vasopressin (0.01–0.03 U/min) is an attractive adjunct, as it causes systemic vasoconstriction while inducing pulmonary vasodilation via nitric oxide stimulation. Phenylephrine should be avoided, as it increases PVR without providing inotropic support [74].
• Inotropic agents: Dobutamine enhances RV contractility and provides modest pulmonary vasodilation, though it may cause dose-dependent tachycardia [75]. Milrinone, a phosphodiesterase-3 inhibitor, provides “inodilator” effects, making it valuable for patients on chronic beta-blocker therapy, though it frequently necessitates concomitant vasopressor support due to systemic vasodilation [76]. Levosimendan, a calcium sensitizer, increases contractility without significantly increasing myocardial oxygen demand and is an attractive option for patients with pulmonary hypertension [77].

### 5.5. Selective Pulmonary Vasodilators

Selective pulmonary vasodilation targets elevated PVR without inducing systemic hypotension. Inhaled nitric oxide (iNO) and inhaled prostacyclins (epoprostenol and iloprost) are particularly advantageous because they are delivered directly to ventilated alveoli, improving ventilation–perfusion matching [78,79]. Inhaled prostacyclins offer a more accessible, lower-cost alternative to iNO with similar efficacy in preventing postoperative RV failure [76]. Systemic pulmonary vasodilators (e.g., sildenafil) have a limited role in the acute phase due to risks of systemic hypotension and unpredictable absorption [80].

### 5.6. Mechanical Circulatory Support

**Figure 3:**
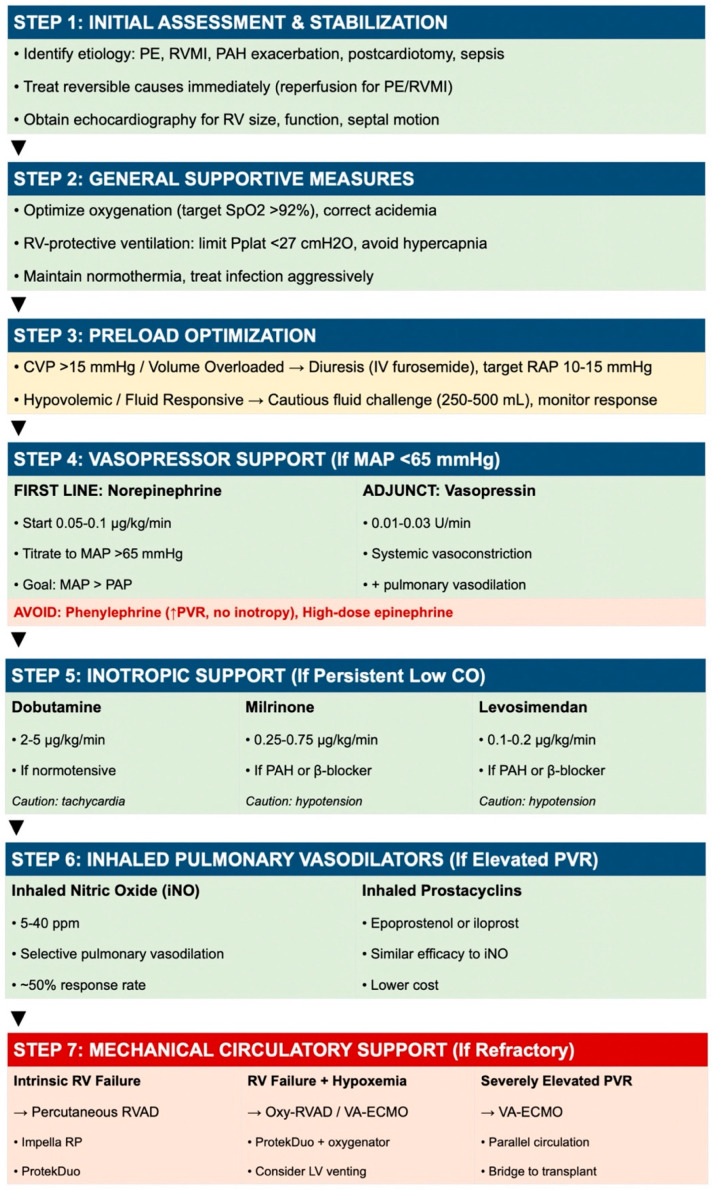
Stepwise management algorithm for acute right ventricular failure. Abbreviations: CO, cardiac output; CVP, central venous pressure; iNO, inhaled nitric oxide; MAP, mean arterial pressure; PAH, pulmonary arterial hypertension; PAP, pulmonary artery pressure; PE, pulmonary embolism; Pplat, plateau pressure; PVR, pulmonary vascular resistance; RAP, right atrial pressure; RV, right ventricle; RVAD, right ventricular assist device; RVMI, right ventricular myocardial infarction; VA-ECMO, venoarterial extracorporeal membrane oxygenation.

When pharmacological therapy fails, MCS provides a critical bridge to recovery or transplantation [81]. The specific device selection logic based on the clinical phenotype is outlined in Figure 3, step 7.

• Direct RV by-pass: The Impella RP (a microaxial flow pump) and ProtekDuo (a dual-lumen cannula) aspirate blood from the RA and expel it into the pulmonary artery, directly bypassing the RV [82].
• VA-ECMO: Provides both circulatory and gas exchange support by establishing a parallel circulation. While effective for patients in extremis, VA-ECMO increases LV afterload, which may require LV venting strategies (e.g., adding an Impella or intra-aortic balloon pump (IABP)) to prevent pulmonary edema.

## 6. Condition-Specific Therapy

### 6.1. Acute Pulmonary Embolism

As a dramatic cause of acute RV failure, risk stratification is essential to guide therapy. High-risk PE mandates immediate reperfusion, but systemic thrombolysis carries significant bleeding risks in patients over 65. Catheter-directed therapies, such as ultrasound-assisted thrombolysis or large-bore aspiration, are vital alternatives that improve the RV/LV ratio while minimizing systemic lytic exposure. Surgical embolectomy remains a Class I recommendation when thrombolysis is contraindicated or fails [21].

### 6.2. Right Ventricular Myocardial Infarction

Recognized in 30–50% of inferior STEMIs, right ventricular myocardial infarction (RVMI) requires management distinct from isolated LV infarction. The classic triad of hypotension, elevated jugular venous pressure (JVP), and clear lungs should prompt immediate right-sided electrocardiographic (ECG) leads (V3R–V4R). Unlike other forms of failure, the RV in RVMI may be genuinely preload-dependent initially, necessitating cautious fluid resuscitation, while nitrates, diuretics, and morphine must be avoided. Early revascularization of the right coronary artery and maintaining atrioventricular synchrony via pacing are critical to preserve the atrial contribution to RV filling.

### 6.3. Pulmonary Arterial Hypertension

Patients with PAH operate at the limits of adaptation; any acute insult can precipitate a life-threatening RV crisis. A paramount principle is the avoidance of abrupt withdrawal of PAH-specific medication, as this can trigger rebound pulmonary hypertension. If oral intake is impossible, transition to parenteral or inhaled prostacyclins must be arranged urgently. Furthermore, intubation and mechanical ventilation are extremely high-risk procedures in this population due to the risk of hemodynamic collapse upon induction; these should be avoided unless absolutely necessary [83].

### 6.4. Postcardiotomy and Post-LVAD Right Ventricular Failure

RV failure occurs in up to 40% of LVAD implantations and remains a major driver of post-surgical morbidity [84]. Management centers on “RV-protective” strategies: optimizing preload, utilizing inhaled pulmonary vasodilators to reduce PVR, and maintaining coronary perfusion. In the post-LVAD setting, the early institution of temporary MCS, such as the ProtekDuo or Impella RP, is increasingly favored over prolonged pharmacological escalation. This “early-exit” strategy from refractory medical therapy is associated with better myocardial recovery and reduced multi-organ failure.

### 6.5. Cardiac Masses

Cardiac masses represent another critical, though less frequent, scenario where RV involvement can have substantial hemodynamic consequences. Masses may lead to right-sided HF through direct inflow or outflow tract obstruction, and they carry a significant risk of pulmonary embolism or, in the presence of a patent foramen ovale, paradoxical systemic embolism [85]. Echocardiography serves as the first-line tool for the initial detection and characterization of these lesions, while CMR holds a central role in distinguishing between malignant tumors, benign masses, and pseudomasses, such as thrombi or prominent normal anatomical variants [86]. While primary cardiac tumors are rare, the RV is more commonly affected by metastatic involvement or the direct extension of renal or hepatic malignancies via the venous circulation. Careful assessment of mass mobility, attachment points, and associated myocardial infiltration is essential for guiding subsequent diagnostic steps and management strategies [87].

## 7. Conclusions

The assessment of RV function in the CCU unit has evolved from a secondary consideration to a primary determinant of patient outcomes. As outlined, the unique physiology of the RV, characterized by a complex anatomy and extreme sensitivity to afterload, requires a specialized approach that prioritizes RV-PA coupling and avoids the pitfalls of aggressive volume loading. By integrating multimodal imaging with precise invasive hemodynamics, such as the PAPi and CVP/PCWP ratios, clinicians can better navigate the complex “vicious cycle” of RV failure. While pharmacological and mechanical support options have expanded significantly, a physiologically informed, multidisciplinary approach remains the cornerstone of care for these high-risk patients.

## 8. Future Directions

Future efforts to improve RV-related outcomes must move beyond expert consensus toward high-quality, evidence-based protocols. This includes the routine clinical integration of advanced metrics such as RV longitudinal strain and 3D echocardiography to detect subclinical dysfunction. Additionally, the application of artificial intelligence must move beyond automated imaging toward advanced predictive modeling using continuous physiological data streams. Integrating these machine-learning algorithms into the electronic health record can provide real-time decision support at the bedside, helping clinicians dynamically differentiate between acute and chronic RV failure phenotypes. This precise phenotyping allows for tailored prognostic information and the highly optimized titration of fluids, inotropes, or MCS. Prospective research focused on validating escalation algorithms for MCS and identifying novel therapeutic targets for myocardial recovery is essential to standardize management and reduce the significant morbidity associated with RV failure. By synthesizing expert consensus with evidence-based protocols and emerging technologies, this review seeks to provide a standardized framework for escalation that supports the clinical management of this high-risk patient population.

## Data Availability

No new data were created or analyzed in this study.

## Abbreviations

The following abbreviations are used in this manuscript:

RV	right ventricle
LV	left ventricle
ACS	acute coronary syndrome
PE	pulmonary embolism
CS	cardiogenic shock
HF	heart failure
CCU	cardiac care unit
AI	artificial intelligence
MCS	mechanical circulatory support
TTE	transthoracic echocardiography
TAPSE	tricuspid annular plane systolic excursion
FAC	fractional area change
TEE	transesophageal echocardiography
RVEF	right ventricular ejection fraction
3D	three-dimensional
CMR	cardiac magnetic resonance
CVP	central venous pressure
PAPi	pulmonary artery pulsatility index
PCWP	pulmonary capillary wedge pressure
CT	computed tomography
STEMI	ST-elevation myocardial infarction
MACE	major adverse cardiovascular events
RA	right atrial
VT	ventricular tachycardia
PESI	pulmonary embolism severity index
PAH	pulmonary arterial hypertension
mPAP	mean pulmonary artery pressure
PVR	pulmonary vascular resistance
AHF	advanced heart failure
LVAD	left ventricular assist device
SVV	stroke volume variation
PPV	pulse pressure variation
IVC	inferior vena cava
iNO	inhaled nitric oxide
IABP	intra-aortic balloon pump
RVMI	right ventricular myocardial infarction
JVP	jugular venous pressure
ECG	electrocardiographic
